# A simulated patient evaluation of pharmacist’s performance in a men’s mental health program

**DOI:** 10.1186/s13104-018-3869-5

**Published:** 2018-10-26

**Authors:** Andrea L. Murphy, David M. Gardner

**Affiliations:** 1 College of Pharmacy, Dalhousie University, 5968 College St., PO Box 15000, Halifax, NS B3H 4R2 Canada; 2 Department of Psychiatry, Dalhousie University, QEII HSC, AJLB 7517, 5909 Veterans’ Memorial Lane, Halifax, NS B3H 2E2 Canada

**Keywords:** Mental disorders, Community pharmacy services, Pharmacists, Simulated patients

## Abstract

**Objective:**

The Headstrong program, a pharmacy based men’s mental health promotion program, was designed to enhance pharmacists’ care of men with mental illness and addictions and was focused on six conditions. A simulated patient (SP) encounter on insomnia was used to evaluate pharmacist’s performance as a part of the Headstrong program.

**Results:**

Six Headstrong pharmacists consented to participate in the SP encounter as part of the evaluation of the Headstrong program. Pharmacists’ mean scores in most categories that were evaluated (e.g., pre-supply/assessment score, sleep score) were lower than expected. In assessing the SP during the encounter, pharmacists’ mean score was 5.7 (SD 2.0) of a possible 13 points. No pharmacists asked about the SP’s age, availability of other supports, allergies, and whether they had an existing relationship with a pharmacist. One pharmacist inquired about medical conditions, and two asked about pre-existing mental health conditions. Three pharmacists inquired about concurrent medications. The Headstrong program was discussed by half of the pharmacists and a resource recommended by the Headstrong program was suggested by one pharmacist. Several pharmacists used self-disclosure as a mechanism to support rapport building. Overall, the SP felt cared for and respected by the pharmacists and had confidence in their knowledge.

## Introduction

Headstrong-Taking Things Head On, hereafter referred to as Headstrong, is a program targeting men in Nova Scotia, Canada, living with mental illness and addictions. The program was launched in Nova Scotia in 23 pharmacies in October, 2017 and was offered in pharmacies for a 6 month period. Headstrong has six areas of focus: suicide, alcohol use, tobacco use, problems with sleep, depression, and anxiety. Headstrong included an education and training program for pharmacists, including a live, face-to-face training session. The pharmacists were also enrolled in an online course, which included modules for the six content areas in addition to modules on motivational interviewing. Pharmacists were provided with signage for their pharmacies to alert existing patients and other customers that pharmacists are a resource that can help with men’s mental health. A website, headstrong.life, was also developed with curated resources including books, apps, and other recommended websites that pharmacists could use and recommend.

A simulated patient (SP) exercise was proposed a priori as part of the Headstrong evaluation following the education and training. Simulated patients have been used as a feasible and useful method for assessing various pharmacist behaviours (e.g., communication skills) and for improving pharmacy personnel’s knowledge and perceptions of their abilities [1–20]. We report on the results of SP encounters as a part of the Headstrong program evaluation.

## Main text

### Methods

We modelled the simulated patient on the work of Kippist et al. [20] and our previous experience [19]. The scenario included a male patient with acute insomnia in their mid-twenties who was a full-time student struggling with insomnia due to final exams. The SP was seeking advice and resources to help overcome their insomnia, with underlying issues of school-related stress and anxiety. One male SP was used for all of the interactions. The SP was instructed to use a standard approach with each pharmacist.

Several categories were evaluated numerically and summarized using descriptive statistics (Table 1). There were additional sections for qualitative comments in the categories of: overall experience, care received, knowledge of the pharmacist, addressing needs, and interaction with other staff. Pharmacists could also provide feedback to the SP after the encounter.

**Table 1:** Simulated patient scoring for pharmacist’s performance in the Headstrong program

Category	Description of variables	Maximum score and number of items
Pre-supply/assessment	Patient’s name, patient age, their personal and/or medical supports, what are the symptoms, duration of the problem, previous occurrence of the problem, current medications, medical conditions, mental health conditions, substance use, whether the patient has a relationship with the pharmacy, any allergies, and past attempts at treatment	13 for 13 items
Headstrong	Mentioned the Headstrong program, explained the Headstrong program, recommended a specific resource from the Headstrong website, provided Headstrong print materials	5 for 5 items
Sleep	Whether the problem was initiating or maintaining sleep, waking unrefreshed, tired throughout the day, sleeping at inappropriate times, triggers and causes, change in sleep environment, whether information was provided on sleeping/insomnia, whether sleep hygiene was discussed, and whether sleep apnea was a concern	10 for 10 items
Guidance	Sleep hygiene guidance provided, specific non-medication resources being promoted.	2 for 2 items
Supply (if sleeping medication provided/advised)	Stating the medications name, dosage information, when to take the medication, expected onset of effect of the medication, duration of use, discussion of side effects, and cost of the medication	7 for 7 items
Communication effectiveness	Empathy, knowledge applied in assessment/treatment, willingness to explore med and non-med resources, organized in assessment, organized in making recommendations, confidence demonstrated in making recommendations, respectful, recommendations easy to understand, asked if clarification was needed, issue was adequately addressed	77 for 11 items ranked from 1 to 7.
Overall rating of quality		10 on a scale from 1 to 10.

The SP was hired through Centre for Collaborative Clinical Learning and Research (C3LR) from Dalhousie University [21].

The Headstrong pharmacists from the 23 Headstrong pharmacies were not required to participate in the SP exercise as part of their participation in the Headstrong program. To meet ethical requirements, consent was required from the pharmacists for the SP activity. All Headstrong program pharmacists were approached by the research coordinator to request participation in the SP activity. Once a pharmacist consented, their schedule for several months in advance and a photo, for easy recognition by the SP, were requested. Pharmacists were not aware of the SP’s visit details (e.g., date and time), appearance, or chief complaint. SP visits occurred at any time between January and March of 2018 and consenting pharmacists were visited once.

For each visit, the SP interacted with pharmacist and then left the pharmacy to complete scoring. The SP then returned that same day to disclose that the deception had occurred and offered to review the information with the pharmacist immediately or at a later time. No further follow-ups with the SP occurred.

### Results

Six pharmacists consented to participating. Following the encounter, all pharmacists agreed to receive and provide feedback immediately.

#### Pre-supply/assessment scores

Pharmacists scored an average of 5.7 (SD 2.0) (Table 2). There were four of 13 variables in which no pharmacists scored points: identifying the patients age, identifying the patients supports, inquiring about any allergies, and inquiring about the patient’s relationship with pharmacists. Categories covered by at least one but fewer than four pharmacists included medications (n = 3), medical conditions (n = 1), mental health conditions (n = 2), and substance use (n = 4).

**Table 2:** Pharmacists’ scores on an acute insomnia simulated patient presentation

Category (maximum possible score)	Mean score (SD)	Minimum–maximum scores
Pre-supply/assessment (13)	5.7 (2.0)	3–9
Headstrong specific (5)	2.2 (2.4)	0–5
Sleep (10)	6 (1.9)	4–8
Guidance (2)	1.3 (0.8)	0–2
Supply (7)	5.2 (0.4)	5–6
Communication effectiveness (77)	65.7 (7.6)	57–75
Overall rating of quality (10)	8 (1.8)	6–10

Five of six pharmacists inquired about the duration of the problem, while all six pharmacists inquired about the patient’s symptoms, the recurrence of the problem, and previous treatments for insomnia.

#### Headstrong specific scores

Of the six pharmacists, three scored four or above (mean 2.2; SD 2.4) (Table 2) points and the remaining achieved zero for Headstrong criteria. Three of the six pharmacists discussed and explained Headstrong and showed or referred the SP to the website. One pharmacist recommended at least one Headstrong resource and two provided them with Headstrong print materials. In general comments, the SP commented that Headstrong promotional materials were at the counter in two stores.

#### Sleep scores

The mean sleep score was 6 (SD 1.9) (Table 2). None of the pharmacists inquired about sleep apnea but all inquired about potential causes of acute insomnia. The same five of six pharmacists inquired about difficulty initiating, difficulty maintaining sleep, and principles of sleep hygiene/non-medication resources. Two pharmacists provided information on sleeping and insomnia.

#### Guidance scores

All but one pharmacist provided guidance on sleep hygiene and three promoted specific non- medication resources for sleep.

#### Supply scores

All pharmacists identified the medication name during medication recommendations, and informed the SP when they should take the medication. Five of six outlined the dosage and duration of use. One pharmacist discussed the cost of the medication.

#### Communication effectiveness

The highest score for communication effectiveness was 75 out of a possible 77 (Table 2). The category with the lowest mean (4.2; SD 2.0) was inquiring about the need for clarification for any advice or recommendations. All pharmacists scored a maximum of 7 points regarding being respectful.

#### Overall rating of quality

The overall quality ratings ranged from six to 10 (Table 2).

#### Qualitative comments by the SP

##### Overall experience in the pharmacy

The SP noted positive experiences overall with pharmacists, feeling that there was an eagerness to help and support them with their sleep problem. They felt comfortable and cared for during the interactions. In two interactions, the SP reported that the availability of Headstrong print materials at the pharmacy counter helped them to initiate the conversation with the pharmacist. The SP noted that in two of the encounters, there were no other customers nearby and in all others, there were one or more customers in the pharmacy area.

##### Level of care received

The SP reported that several pharmacists directed the SP to an aisle with over the counter medication options for sleep to discuss the nature and duration of their sleep problem, triggers, and to suggest solutions. Pharmacists recommended various options, including over-the-counter medication, sleep hygiene practices, and Headstrong materials. In one instance, the pharmacist emphasized the importance of connecting with their family doctor if their sleep issues continued, which made the SP feel cared for and supported. Some pharmacists took the time to understand the underlying causes of the SP’s sleeping problems, and suggested solutions with their unique circumstances as a student in mind.

##### Pharmacists’ level of knowledge

The SP felt confident in the knowledge of each pharmacist. Pharmacists often kept the patient’s circumstances at the forefront of their discussion and recommendations, to ensure the solutions being presented would not negatively affect the SP’s schooling. On some occasions, the SP noted the interactions could have been improved by the pharmacists “digging deeper” to learn more about the patients’ history with sleep problems and/or high levels of stress, and the overall duration of the problem. Resources and information provided to the SP included sleep efficiency information, sleep hygiene practices, a hard-copy sleep journal, and more than one over-the-counter medication option, both for short-term and long-term solutions.

##### Addressing patient needs

The SP felt that their sleep needs were addressed in all interactions. The SP commented on the appreciation for the empathy expressed by pharmacists. In some cases, the SP indicated that they would have preferred more exploration in terms of non-medicinal solutions, such as sleep hygiene. In addition, the SP hoped that more pharmacists would have taken the time to explore the underlying cause of insomnia (i.e., stress).

##### Additional comments by the simulated patient

The SP valued pharmacists’ techniques to build rapport including humour, self-disclosure, and making an effort to learn more about them (e.g., inquiring regarding area of study). These efforts made the SP feel more comfortable. The SP noted appreciation for the discussion of costs on a student’s budget. For example, one pharmacist noted the higher cost of some subscription-based cognitive behavior therapy for insomnia resources on the Headstrong website.

#### Participating pharmacists’ comments

Pharmacists thought the SP case flowed naturally. One pharmacist reported, “The scenario was believable, [and] something I would encounter in my day-to-day practice” (pharmacist 2). Pharmacist 3 also reported that it “wasn’t my best work” and explained their unique circumstances that hindered performance. This included issues of short-staffing, which focused the pharmacist’s time and efforts on preventing prescription-related errors in the dispensary. It was, however, reported as a valuable learning opportunity with appreciation of the feedback. Another pharmacist appreciated the feedback on their lack of emphasis on non-medicinal resources and supports for insomnia.

### Discussion

Using SP encounters to measure pharmacists’ performance in a men’s mental health promotion program was useful to identify potential areas of improvement for pharmacists regarding their assessment and management of acute insomnia. More than half of the pharmacists promoted nonpharmacological supports for insomnia including, but not limited to, Headstrong resources, which was emphasized during the Headstrong education and training. Some areas such as inquiring about concurrent medical conditions, medications, mental health conditions, and allergies were not assessed as would have been expected. This finding concurs with existing literature. In a study on pharmacists’ recommendations in Jordan for over the counter medications for headache, medication recommendations were frequently made without obtaining essential information about the SP’s symptoms or medical history [18]. This also occurred in a community pharmacy SP study in Australia with smoking cessation [1]. In a previous telephone-based insomnia SP study, 35% of the intervention versus 10% of control group pharmacists inquired about medical conditions [19]. Our findings in context with the broader literature in this area suggest that pharmacists assessing patients without adequate knowledge of their medical conditions may lead to harmful recommendations. This could occur through several mechanisms. For example, failing to rule out whether an underlying condition could be exacerbating or causing the presenting illness may lead to unnecessary use of a medication, which inherently may have adverse effects. Recommending a medication without knowledge of underlying disease processes may also put the patient at risk for harm if a medication were to worsen symptoms of the disease.

In our study, all pharmacists asked regarding previous treatments for insomnia, yet half inquired about concurrent medication use. Issues with pharmacists acquiring incomplete medication histories have been reported by others. A study of SPs obtaining prescription medications for diabetes and asthma in Qatar demonstrated that fewer than 4% of pharmacists inquired regarding concurrent medications [4]. Similarly in the smoking cessation study in Australia by Saba et al. [1], fewer than 5% of the simulations with a pregnant woman were asked about concurrent medications. For the second scenario regarding an older adult with cardiovascular disease who was attempting to quit smoking, fewer than one in four of the encounters included questioning about concurrent medications [1]. In the telephone simulation regarding insomnia, more pharmacists in the intervention group (62%) compared to the controls (48%) asked the SP regarding their concurrent medications [19]. Although 69% of SPs with gastroesophageal reflux symptoms presenting to community pharmacists in Australia had an adequate medication history taken, three in 10 did not before pharmacists made recommendations [6]. These findings have important ramifications given the potential for pharmacodynamic and pharmacokinetic drug–drug interactions that can occur when combining treatments.

For medication recommendations, all pharmacists in our study discussed the name and when to take it, but not all discussed the dose, the expected onset of effect, how long to use it, side effects, and only one pharmacist discussed cost. Saba et al. similarly reported that the majority of encounters for smoking cessation products lacked information given by the pharmacist such as when to take the medication, adverse effects, duration of therapy, and how to use the products [1]. These findings are important from a shared decision making perspective as many patients prefer and value getting this type of information (e.g., duration of treatment) to inform their treatment choices [22].

## Limitations

- Six pharmacists from the 23 participating pharmacies in the Headstrong program consented to participation in the SP activity.
- Pharmacists may have changed their behaviour knowing that a SP visit was possible during the 3 month observation period.
- There was no control group and the pharmacists were not randomized to receive an SP visit.
- We used existing literature and our own experience to develop the standardized patient case and assessments, which were not validated.
- The SP encounters were not video or audio recorded. The data analysis was conducted on information transcribed from what the SP recorded immediately following the encounters.

## Abbreviation

SP: simulated patient
